# Clinical and cephalometric comparison: TMA versus AJ wilcock MBT intrusion arches

**DOI:** 10.6026/973206300223539

**Published:** 2026-06-30

**Authors:** Shivani Gupta, Purva Joneja, Richa Sharma, Sachin Jayaraj V., Ulrika Diana Pereira Kalia, Rishabh Joneja

**Affiliations:** 1 Department of Orthodontics and Dentofacial Orthopedics, Bhabha College of Dental Sciences, Bhopal, India; 2 Department of Dentistry, Gandhi Medical College, Bhopal, India; 3 Department of Orthodontics and Dentofacial Orthopedics, Pacific Dental College and Research Centre, Udaipur, Rajasthan, India; 4 Department of Orthodontics and Dentofacial Orthopedics, 1st Faculty of Medicine, Charles University, Prague, Czech Republic

**Keywords:** AJ Wilcock wire, deep bite, incisor intrusion, ricketts utility arch, titanium molybdenum alloy (TMA) wire

## Abstract

Deep bite is one of the most challenging malocclusions to correct orthodontically, yet the comparative effectiveness of available wire materials for intrusion arch fabrication remains insufficiently studied. Therefore, it is of interest to assess the rate of maxillary incisor intrusion achieved with each wire type over a defined period, analyzing the efficiency of force application and biomechanical properties. Ten patients in the age group of 15-30 years were assigned to two groups: Group A (0.017" x 0.025" TMA wire) and Group B (0.018" Special + AJ Wilcock wire). Clinical and radiographic evaluations were conducted at baseline (T0) and after 3 months of active intrusion (T2). Thus, data shows the understanding of AJ Wilcock wire's potential in improving deep bite correction outcomes in specific orthodontic cases.

## Background:

Orthodontic treatment frequently involves addressing deep overbites, a common malocclusion characterized by excessive vertical overlap of the maxillary and mandibular incisors [1]. Correction strategies vary based on the patient's diagnosis and skeletal pattern, often involving incisor intrusion, posterior extrusion, or a combination of both to achieve a stable functional and esthetic outcome [2]. Orthodontic intrusion involves the controlled apical movement of a tooth's centroid relative to the occlusal plane, requiring precise biomechanical control to minimize side effects like root resorption or unintended tipping [3]. Various mechanics have been used for this purpose, including the ricketts utility arch, Connecticut intrusion arch, mini-implant-supported techniques [4]. Utility arch using a 0.016 x 0.016-inch blue elgiloy wire to provides efficient, predictable intrusion while managing anchorage independently from the posterior segments. In contemporary practice, titanium molybdenum alloy (TMA) and AJ wilcock (Australian) wires are frequently used as alternatives [5]. TMA is valued for its high spring-back, low stiffness and excellent formability, while AJ Wilcock wire offers exceptional flexibility, resiliency and shape memory. Despite their widespread use, there is a lack of research specifically evaluating and comparing the effectiveness of these two materials when used in the construction of utility arches for maxillary deep bite correction [6]. Therefore, this study is important to assess evaluate the rate of intrusion, treatment duration and patient comfort between these wire types.

## Methodology: 

Ten patients were selected after ethical and consent clearance based on inclusion criteria: Age group of 15-30 years, Angle's Class I or Class II malocclusion and a deep bite ranging from 50-100% following initial alignment, optimal range of U1-NA (17-240). Exclusion criteria included syndromic patients, skeletal asymmetry, history of trauma to maxillary incisors, or previous endodontic treatment of anterior teeth.

Group Allocation the subjects were divided into two groups of five subjects each:

[1] Group A (Control Group): Intrusion arch fabricated with 0.017" x 0.025" TMA wire.

[2] Group B (Study Group): Intrusion arch fabricated with 0.018" Special+ AJ Wilcock wire.

Intrusion arches of Group A and Group B were fabricated using the Robert M. Ricketts Utility arch design. Initial leveling and alignment were achieved using a sequence of NiTi archwires. The ricketts design utility arches were then inserted into molar tubes and incisor brackets, bypassing premolars and canines, with a 30° gable bend placed at the junction of the molar and anterior segments. Initial activation delivered 15-20 grams of force per incisor (total 60-80 g), calibrated using a Dontrix gauge. Reactivations occurred at intervals of 21-25 days over a 3-month period. Posterior anchorage was reinforced with a 0.019" x 0.025" stainless steel base arch or a transpalatal arch (TPA) where necessary. Pre-treatment and Post-Treatment Intraoral Photographs, Study Models, Lateral cephalograms were obtained at three stages: T0 (Baseline), T1 (6 weeks) and T2 (3 months' post-active intrusion), along with clinical evaluation. Clinical Records: Intraoral photographs and study casts were used to measure overbite correction and crown length. Radiographic Records: Pre-treatment and post intrusion Lateral cephalograms were obtained to measure dental parameters (U1-Palatal Plane, U1-SN, U1-NA, Overbite, Overjet) and skeletal parameters (Anterior/Posterior Facial Heights and facial ratios). Overbite was measured with the patient in maximum intercuspation using a digital Vernier caliper (accuracy 0.01 mm) at various intervals after initial leveling and alignment at T0, T1, T2 and T3. Measurement was taken from the incisal edge of the maxillary central incisor to the incisal edge of the mandibular central incisor parallel to the long axis of the lower incisor.

## Bite correction was calculated as:

Overbite Reduction (mm) = Overbite at T0 - Overbite at T2

Percentage Reduction = (Overbite reduction / Overbite at T0) x 100

Study casts were also used for verification of measurements.

Cephalometric analysis was performed manually using acetate tracing sheets and standard orthodontic tools. Pre-treatment lateral cephalogram was callibrated to confirm the indication and extent of required true intrusion. Post intrusion lateral cephalogram was taken after 3 months of the same patient and comparison of results was studied. Statistical analysis was conducted using SPSS software version 22, utilizing tests such as the Unpaired Student t-test for group comparisons, Chi-Square test and ANOVA. All data were analyzed using IBM SPSS Statistics version 30. Normality of continuous variables was checked using the Shapiro-Wilk test and Q-Q plots and all parameters were approximately normally distributed. Paired t-tests were used to compare pre- and post-treatment values within each group, while independent t-tests were used to compare mean changes between the TMA and AJ Wilcock groups. Repeated measures ANOVA was used to assess changes in bite depth and crown length over time, with time x group interactions examined to detect differences in treatment effects between groups. Effect sizes were calculated using Cohen's d and Hedges' g and p < 0.05 was considered statistically significant. Overall, linear, dental and clinical parameters showed significant improvement after treatment, with no significant differences between TMA and AJ Wilcock wires, indicating equivalent clinical effectiveness.

## Results:

An independent samples t-test was performed to compare linear cephalometric changes between the TMA wire group (n = 5) and the AJ Wilcock wire group (n = 5). No statistically significant differences were observed between the two groups for maxillary incisor intrusion (U1-PP: mean difference = 0.40 mm; t = 1.414; p = 0.195), molar extrusion (U6-PP: mean difference = 0.10 mm; t = 0.343; p = 0.740), or anterior facial height (AFH: mean difference = -0.20 mm; t = -0.632; p = 0.545). Effect size analysis revealed moderate magnitude for incisor intrusion (Cohen's d = 0.894; Hedges' g = 0.807) and small effect sizes for molar extrusion and AFH, suggesting minimal clinical differences between groups. Posterior anchorage control, assessed through upper first molar extrusion (U6-PP), showed a mean of 0.50 ± 0.50 mm for TMA and 0.40 ± 0.42 mm for AJ Wilcock, with a negligible mean difference of 0.10 mm. The t-value of 0.343 and p-value of 0.740 confirmed that this difference was far from statistically significant. Supporting this, Cohen's d of 0.217 and Hedges' g of 0.196 both indicate a small effect size, suggesting that the two wire types performed virtually identically in terms of posterior anchorage. This finding is of considerable clinical relevance, as uncontrolled molar extrusion represents an unwanted side effect of intrusion mechanics that would not only counteract deep bite correction but also increase anterior facial height. The comparable and minimal molar extrusion observed with both wires therefore confirms that either material can be reliably employed without compromising anchorage integrity during active intrusion therapy. Changes in anterior facial height (AFH) were 0.60 ± 0.55 mm for TMA and 0.80 ± 0.45 mm for AJ Wilcock, with a mean difference of -0.20 mm, indicating that AJ Wilcock produced a marginally greater increase in vertical facial dimension. The t-value of -0.63 and p-value of 0.545 confirmed this difference to be statistically non-significant, while Cohen's d of -0.400 and Hedges' g of -0.361 placed the effect in the small-to-medium range. The negative sign of the effect size reflects AJ Wilcock's slightly greater tendency toward vertical facial height increase, though the absolute magnitude of less than 1 mm over three months renders this clinically inconsequential for most patients. Maintenance of vertical dimension is a critical consideration in deep bite management, as excessive anterior facial height increase can destabilize treatment outcomes. Both wires demonstrated commendable vertical control within this timeframe, supporting their equivalence in preserving facial height stability (Table 1). An independent samples t-test was conducted to compare angular changes in maxillary anterior teeth between the TMA and AJ Wilcock wire groups (n = 5 per group). Among the evaluated parameters, a statistically significant difference was observed only for UI-PP (°), where the AJ Wilcock group demonstrated greater angular change compared to the TMA group (mean difference = -1.00°; t = -2.77; p = 0.024). The effect size was large (Cohen's d = -1.754; Hedges' g = -1.58), indicating a substantial difference in incisor inclination relative to the palatal plane. Similar proclination tendencies during intrusion mechanics have been previously reported in comparative intrusion studies [6, 7]. For the remaining angular parameters PP-SN, OP-SN, OP-PP, U1-NA and U1-SN, no statistically significant differences were detected between the groups (p > 0.05). Regarding angular skeletal and dental changes, the palatal plane angle relative to the cranial base (PP-SN) showed a mean change of 0.80 ± 0.45° in the TMA group and 1.30 ± 0.45° in the AJ Wilcock group, with a mean difference of -0.50°. The t-value of -1.76 and p-value of 0.115 indicated no statistically significant difference between the groups, yet Cohen's d of -1.118 and Hedges' g of -1.00 both reflect a large effect size, suggesting a clinically meaningful trend toward greater palatal plane rotation in the AJ Wilcock group that the study was likely underpowered to confirm statistically. Similarly, the occlusal plane angle relative to the cranial base (OP-SN) demonstrated mean changes of 1.30 ± 0.45° and 1.80 ± 0.57° for TMA and AJ Wilcock respectively, yielding a mean difference of -0.50°. Despite a t-value of -1.54 and a non- significant p-value of 0.161, Cohen's d of -0.976 and Hedges' g of -0.88 again indicate a large effect size, reinforcing the pattern of a possible but statistically unconfirmed tendency for greater angular change with AJ Wilcock wire. In contrast, the occlusal plane angle relative to the palatal plane (OP-PP) showed negligible differences, with mean changes of 1.00 ± 1.00° and 1.10 ± 0.42 ° for TMA and AJ Wilcock respectively, a mean difference of only -0.10°, a t-value of -0.20, p-value of 0.842, and a trivially small effect size (Cohen's d = -0.130, Hedges' g = -0.117), collectively confirming true equivalence between the two wires for this parameter. Among the dental angular measurements, the U1-NA angle showed mean changes of 5.20 ± 0.84° and 5.60 ± 0.89° for TMA and AJ Wilcock respectively, with a mean difference of -0.40°, a t-value of -0.73, and a non-significant p-value of 0.486. The small-to- medium effect size (Cohen's d = -0.462, Hedges' g = -0.41) suggests minimal practical difference between the two wire types for this measure. The U1-SN angle demonstrated mean changes of 5.10 ± 0.65° for TMA and 6.00 ± 0.79° for AJ Wilcock, with a mean difference of -0.90° and a p-value of 0.085 that narrowly missed statistical significance. However, Cohen's d of -1.242 and Hedges' g of -1.12 indicate a large effect size, representing another instance where the trend toward greater angular change with AJ Wilcock wire approached but did not reach the conventional threshold of significance, most plausibly due to inadequate statistical power from the limited sample size. The most clinically and statistically compelling finding in this parameter set was the upper incisor inclination relative to the palatal plane (UI-PP), which showed mean changes of 3.80 ± 0.57° in the TMA group and 4.80 ± 0.57° in the AJ Wilcock group, yielding a statistically significant mean difference of -1.00° (t = -2.77, df = 8, p = 0.024). This was the only parameter in the entire table to reach statistical significance, and the effect size was correspondingly very large (Cohen's d = -1.754, Hedges' g = -1.58), indicating that AJ Wilcock wire produced substantially greater incisor inclination change relative to the palatal plane compared to TMA wire. This finding suggests that while both wires achieve comparable levels of incisor intrusion, AJ Wilcock wire exerts a greater proclinatory or torque-related effect on the maxillary incisors, which carries important implications for treatment planning in cases where incisor angulation control is a priority (Table 2). At baseline (T0), the TMA group presented with a mean bite depth of 4.164 ± 0.238 mm, while the AJ Wilcock group showed a slightly greater initial bite depth of 4.600 ± 0.588 mm, yielding a combined total mean of 4.382 ± 0.481 mm. By the first follow-up (T1), both groups demonstrated measurable reduction, with TMA recording 3.470 ± 0.192 mm and AJ Wilcock recording 3.960 ± 0.602 mm, reflecting a total mean of 3.715 ± 0.494 mm. This downward trajectory continued at T2, where TMA showed a mean bite depth of 2.920 ± 0.164 mm and AJ Wilcock recorded 3.400 ± 0.543 mm, with a combined mean of 3.160 ± 0.455 mm. By the final time point (T3), both groups achieved their lowest recorded bite depths, with TMA reaching 2.324 ± 0.043 mm and AJ Wilcock reaching 2.980 ± 0.559 mm, producing a total mean of 2.652 ± 0.509 mm (Table 3). Repeated measures ANOVA demonstrated a statistically significant reduction in bite depth over time in both TMA and AJ Wilcock groups (F = 261.673, p < 0.001), with a very large effect size (Partial Eta^2^ = 0.970), indicating substantial treatment-related improvement from T0 to T3. However, the Time x Group interaction was not statistically significant (F = 1.104, p = 0.367), suggesting that the pattern of bite depth reduction over time was comparable between the two wire systems. This pattern of progressive vertical reduction is comparable to clinical outcomes reported in deep bite correction studies. Additionally, the between-subjects effect of Group was not statistically significant (F = 4.055, p = 0.079), indicating no overall difference in mean bite depth reduction between TMA and AJ Wilcock wires. These findings confirm that both intrusion arches were equally effective in reducing bite depth over the study period, with similar treatment progression and clinical outcomes (Table 4).

## Discussion:

In orthodontics, correcting deep overbite by actual maxillary incisor intrusion is still a biomechanically delicate procedure. The current study used TMA and AJ Wilcock intrusion arch mechanics to assess the skeletal and dental changes that occur after true intrusion. The results showed a statistically significant decrease in U1-SN and U1-PP values, suggesting minimal proclination and effective true intrusion with both TMA and AJ Wilcock type of intrusion arches [7]. In the literature, intrusion mechanics have been thoroughly explained. Light continuous forces (10-20 g per incisor) are crucial for attaining pure intrusion while reducing root resorption. Because considerable vertical reduction was accomplished without undesired tipping movements, the current study's findings are consistent with Burstone's biomechanical principles [8]. According to Karanth and Shetty comparative study [9] on intrusion made off with various alloys, determines the optimal intrusion force exerted by utility arches made with 0.016 and 0.018 Australian special+wires. The study suggests that the high resilience of the Australian wire, when designed in the form of a Ricketts Utility Arch, can be effectively used for true maxillary incisor intrusion. This approach facilitates significant and rapid bite opening (2.3 mm) while minimizing undesirable labial flaring of the maxillary incisors fall within these ranges. It is particularly indicated in cases where the U1-NA Linear measurement ranges from 3-5mm and the U1-NA Angular value lies between 17° and 24°, which represent ideal incisor positioning relative to the NA line. Nonetheless, TMA intrusion arches showed comparatively better torque control and a decreased proclination tendency, indicating greater biomechanical efficiency in preserving incisor inclination during intrusion [10]. The current study offers quantitative cephalometric evidence demonstrating the effectiveness of TMA intrusion mechanics in achieving controlled true intrusion with negligible side effects, whereas prior literature primarily concentrates on biomechanical concepts or temporary dental changes. The mechanotherapy used did not negatively impact vertical facial proportions, as evidenced by the absence of any notable undesired skeletal changes, such as an increase in anterior facial height or mandibular plane angle for both the groups. According to earlier research, intrusion mechanics may change the height of the anterior face and the occlusal plane. According to Arnett and Bergman [11] if posterior extrusion is managed, pure incisor intrusion shouldn't have a major impact on anterior facial height. The mechanics mostly produced true intrusion rather than relative intrusion through molar extrusion, as evidenced by the current study's shows no or negligible increase in anterior facial height. In terms of angular changes, our study's finding of a decrease in the U1-SN angle suggests better axial inclination after intrusion. The anchorage control procedure used in our investigation could be the cause of the discrepancy. During intrusion, root resorption is still a concern. Dermaut and Munck (1986) [12] showed that the risk of apical root resorption is increased by excessive intrusive forces. Despite the fact that radiographic evaluation was not the main goal of this investigation, no clinical indications of pathological resorption were found, most likely as a result of the application of light continuous forces. According to Topkara *et al.* (2012) [13] biomechanical theories, the results clinically support the use of light continuous force systems to achieve controlled incisor intrusion. The current study offers recommendations for choosing suitable intrusion mechanics in deep bite correction and supplements the body of existing literature with comparative cephalometric evidence. To assess long-term stability and root integrity after true intrusion, more longitudinal research with bigger sample sizes and three-dimensional imaging is advised. Furthermore, as our study utilized a controlled sample of ten post-pubertal patients, there is a clear need for future research with larger sample sizes and long-term follow-up to establish more definitive findings on the stability of this correction.

## Conclusion:

The 0.018" AJ Wilcock Special+ wire is a viable and cost-effective alternative to TMA for true maxillary incisor intrusion when used in properly selected deep overbite cases with favorable baseline cephalometric values. Its use should be based on careful diagnosis and treatment planning, as it is not meant for routine or indiscriminate application in all intrusion cases. Further research is needed to evaluate its mechanics, force levels, intrusion rate and possible root resorption, particularly in the Central Indian population using advanced imaging such as CBCT.

## Figures and Tables

**Table 1:** Comparison of linear cephalometric changes between TMA and at wilcock wires (n=10)

**Parameter**	**Groups (Mean ± SD)(mm)**		**Mean Difference**	**t**	**df**	**p-value**	**Cohen's d**	**Hedges' g**
	**TMA**	**AJ Wilcock**						
U1-PP (Incisor intrusion)	2.70 ± 0.45	2.30 ± 0.45	0.4	1.414	8	0.195 (NS)	0.894	0.807
U6-PP (Molar extrusion)	0.50 ± 0.50	0.40 ± 0.42	0.1	0.343	8	0.740(NS)	0.217	0.196
AFH (Anterior Facial Height)	0.60 ± 0.55	0.80 ± 0.45	-0.2	-0.63	8	0.545(NS)	-0.4	-0.361

**Table 2:** Comparison of angular changes in maxillary anterior teeth between TMA (Group 1) and AJ Wilcock wire (n-10)

**Parameter**	**TMA Mean ± SD**	**AJ Wilcock Mean ± SD**	**Mean Difference**	**t**	**df**	**p-value**	**Cohen's d**	**Hedges' g**
PP-SN (°)	0.80 ± 0.45	1.30 ± 0.45	-0.5	-1.76	8	0.115	-1.118	-1
OP-SN (°)	1.30 ± 0.45	1.80 ± 0.57	-0.5	-1.54	8	0.161	-0.976	-0.88
OP-PP (°)	1.00 ± 1.00	1.10 ± 0.42	-0.1	-0.2	8	0.842	-0.13	-0.1
U1-NA (°)	5.20 ± 0.84	5.60 ± 0.89	-0.4	-0.73	8	0.486	-0.462	-0.41
U1-SN (°)	5.10 ± 0.65	6.00 ± 0.79	-0.9	-1.96	8	0.085	-1.242	-1.12
UI-PP (°)	3.80 ± 0.57	4.80 ± 0.57	-1	-2.77	8	0.024*	-1.754	-1.58
*Statistically significant at p <
0.05, NS- Nonsignificant

**Table 3:** Comparison of mean bite depth (mm) changes over time between TMA and at wilcock wires with repeated measures ANOVA

**Time**	**TMA (Mean ± SD)**	**AJ Wilcock (Mean ± SD)**	**Total (Mean ± SD)**
T0	4.164 ± 0.238	4.600 ± 0.588	4.382 ± 0.481
T1	3.470 ± 0.192	3.960 ± 0.602	3.715 ± 0.494
T2	2.920 ± 0.164	3.400 ± 0.543	3.160 ± 0.455
T3	2.324 ± 0.043	2.980 ± 0.559	2.652 ± 0.509

**Table 4:** Repeated measures ANOVA Results

**Effect**	**F (df)**	**p-value**	**Partial Eta^2^**
Time	261.673 (3)	<0.001*	0.97
Time x Group	1.104 (3)	0.367	0.121
Group	4.055 (1)	0.079	0.336
*Statistically significant
at p < 0.05
